# The relationship between abdominal aortic aneurysm diameter and its risk factors: a retrospective cohort study

**DOI:** 10.1590/1677-5449.202301102

**Published:** 2025-02-14

**Authors:** João Victor Domiciano Martins, Rodrigo Mendes, Ronald Luiz Gomes Flumignan, Luiz Carlos Uta Nakano, Jorge Eduardo de Amorim, Henrique Jorge Guedes

**Affiliations:** 1 Universidade Federal de São Paulo – UNIFESP, São Paulo, SP, Brasil.; 2 Irmandade da Santa Casa de Misericórdia de São Paulo – FCMSCSP, São Paulo, SP, Brasil.

**Keywords:** abdominal aortic aneurysm, risk factors, diameter

## Abstract

**Background:**

Abdominal aortic aneurysm is defined as a focal and persistent dilatation of the abdominal portion of the aorta to a diameter ≥50% larger than the diameter of adjacent segments and involving all three layers of the vessel wall.

**Objectives:**

To evaluate whether risk factors (diabetes mellitus, hypertension, dyslipidemia, smoking, and age) influence aneurysm expansion.

**Methods:**

This is a retrospective observational study of a series of cases that included 299 patients treated from January 2007 to January 2020, separated into exposed and unexposed groups by risk factors. Student’s *t* test was used to assess whether mean aneurysm diameters showed statistically relevant differences between groups. A multivariate regression analysis was also conducted with the same groups.

**Results:**

Smokers had larger aneurysms than those who had never smoked (p=0.002) and than former smokers (p<0.01) and patients ≤65 years old had larger diameters compared to patients aged 66 to 75 years old (p=0.0226). There were no significant correlations with the other risk factors (diabetes mellitus, dyslipidemia, hypertension). Multivariate regression analysis confirmed the same result, but with a coefficient of determination of 0.0608. Furthermore, smokers, patients with hypertension, patients with dyslipidemia, and patients without diabetes had higher frequencies of much larger aneurysm diameters.

**Conclusions:**

It was observed that age ≤65 years and current smoking were related to greater aneurysm diameter. In contrast, the same statistical relationship was not observed for hypertension, absence of diabetes, or dyslipidemia, since there was a greater frequency of discrepant values for these groups. Studies are needed with a more comprehensive analysis of determinants of aneurysm diameter.

## INTRODUCTION

Abdominal aortic aneurysm (AAA) is a multifactorial degenerative disease^1-3^ with approximate incidence of 5% of the population without comorbidities, becoming more common with aging and presence of comorbidities such as atherosclerosis, systemic arterial hypertension (SAH)^4-8^ and connective tissue and collagen diseases.^9-11^ Moreover, there are notable associations between incidence of aneurysms and smoking, inactivity, and a high fat diet.^7,8,12-15^ Diabetes mellitus (DM) is not linked to a higher risk of AAA,^16^ although there are contradictory findings in the literature and it has previously been considered a protective factor.^12^ Moreover, some recent studies suggest that metformin plays a role in reducing the prevalence and extent of aneurysm sac expansion.^17^ Approximately, 175,000 deaths per year are attributed to ruptured AAAs.^18^

Aortic aneurysm can be defined as a dilatation to a diameter more than 50% larger than the natural diameter of the aorta.^19^ Dilatation is caused by inflammatory destabilization of the tunica media, which loses elasticity and resistance because of an inflammatory phenomenon known as cystic medial degeneration.^20-22^ Changes to the camara media, associated with pulsatile blood flow and high pressure, lead to gradual and concentric dilatation of the aorta, forming an aneurysm.^19,23-25^

AAA can manifest clinically (with mild symptoms or, in the majority of cases, as an examination finding^17^ ) or as an emergency, presenting with abdominal pains, hemorrhagic acute abdomen, or hypovolemic shock, when the aneurysm ruptures.^19,26,27^ Diagnosis can be with ultrasonography, magnetic resonance, or angiotomography (the gold standard).^19,28^ Treatment varies depending on the clinical presentation: for cases with low risk of rupture, regular follow-up is recommended in combination with treatment for the underlying diseases and control of risk factors. Surgical repair is indicated if the dilatation exceeds 5 cm in women and 5.5 cm in men, and also in cases of rapid expansion of the aneurysm.^29^ When urgent, treatment is founded on control of shock and surgery.^29^

Surgery can be performed with open techniques, with resection of the aneurysmal segment of the aorta and substitution with vascular graft, or using endovascular techniques, with placement of endoprostheses.^30,31^ Countless complications can occur, depending on the surgical technique adopted, the surgeon’s experience, aneurysm site, length, and other factors, and individual management is necessary.^32-34^

The estimated prevalence of AAA in the population aged > 50 years in the city of São Paulo varied from 1.9 to 2.96%.^34^ According to the Brazilian Institute of Geography and Statistics (IBGE - Instituto Brasileiro de Geografia e Estatística) (IBGE), in 2010, the city of São Paulo had approximately 6.8 million inhabitants aged > 50 years. On that basis, the number of people in that age group who had AAA was in the range of 129,000 to 201,000.

In order to define public health policies, it is important to know the prevalence of aortic aneurysms in a given population and its subsets in order to prioritize high risk populations. The objective of this study was to determine the statistical relevance of risk factors for expansion of AAAs in a group of patients at a University Hospital in the city of São Paulo. The article was written in accordance with the Strengthening the Reporting of Observational studies in Epidemiology (STROBE) guidelines for cohort studies.

## MATERIALS AND METHODS

### Data sources

This retrospective cohort study employed data from a database of digital medical records from a tertiary level teaching hospital in the largest metropolis in Latin America. The study was evaluated and approved by the hospital’s Ethics Committee, with Ethics Appraisal Submission Certificate 46678320.3.0000.5505 and Consolidated opinion number 4.869.330.

### Selection of patients

**Figure 1:**
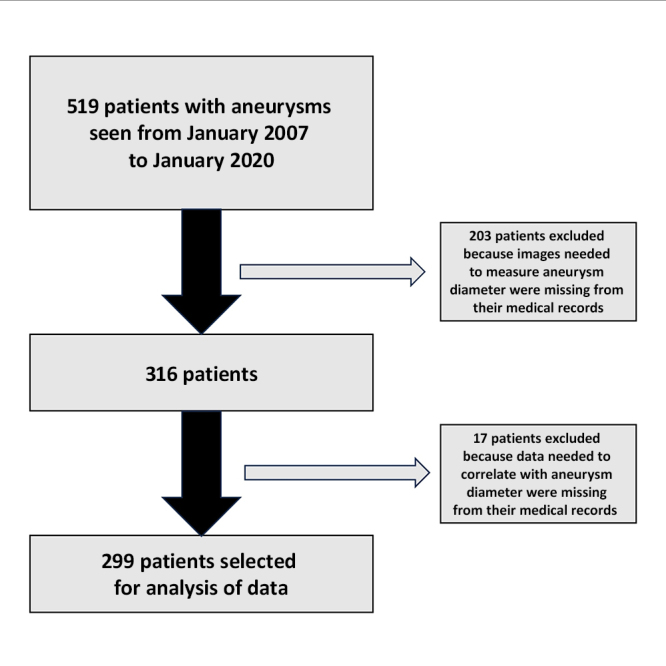
Flowchart illustrating inclusion and exclusion of patients. A total of 519 patients were admitted with abdominal aortic aneurysm from January 2007 to January 2020. Of these, 203 were excluded because it was not possible to determine the diameter of their aneurysms and 17 were excluded because data were missing from their electronic medical records on the risk factors analyzed for this study.

Data were extracted from the medical records of patients with indications for surgical AAA repair admitted to the Vascular and Endovascular Surgery Department at the Hospital São Paulo from January 2007 to January 2020. Surgical indications were searched to identify patients with ruptured aneurysms, symptomatic aneurysms, with rapid growth in aneurysmal diameter (0.5 cm in 6 months or 1 cm in 1 year), saccular aneurysms, and aneurysms with large diameters (> 5 cm for women and > 5.5 cm for men). However, 220 electronic patient records were excluded because it was not possible to access images to measure aneurysm diameter and/or because of lack of sufficient clinical data to conduct analyses for the study. This occurred because some of the medical records from 2007 to 2011 had not been correctly imported into the hospital’s electronic system, resulting in loss of 65.625% of the cases from that period, whereas losses of cases from 2012 onwards equated to 13.41% of the total number of medical records (7.35% losses because of missing information on the risk factors analyzed and 6.06% because of inability to access images to measure aneurysm diameter). Additionally, the process for requesting physical medical records would have logistically prevented the study from being completed in time, because the process for obtaining authorization and requesting these medical records would take at least 6 months. As a result, 299 medical (or patient) records were selected and analyzed for this study (Figure 1).

### Covariates and results

The following independent variables were obtained from patients’ medical records to determine the relevance of risk factors: presence of DM, SAH, and dyslipidemia (DLP), presence of smoking, and age group (≤ 65 years, from 66 to 75 years, > 75 years). The dependent variable in the analysis was the diameter of the aneurysm sac, determined at the largest diameter of the infrarenal abdominal aorta, perpendicular to blood flow, and measured on abdominal CT images accessed via Synapse, which is software for viewing imaging exams embedded in the electronic medical record system.

Additionally, the following factors were tabulated to trace the profile of the hospital’s patients: sex, occupation, surgical method (open or endovascular), and place of origin/nationality.

### Statistical analysis

The data obtained from medical records were tabulated using Excel and imported into SPSS, where AAA diameters (dependent variable) were correlated with presence of associated risk factors (independent variables), to establish statistically whether specific risk factors were relevant to increased progression of the aneurysm.

For each risk factor, patients were classified into two groups. For each group, mean diameter was calculated and the Levene test was applied, to compare variances and obtain an initial p value. If p > 0.05, it was assumed that variances were homogeneous (hypothesis H_0_) and if p < 0.05, it was assumed that variances were not homogeneous (hypothesis H_1_).

After obtaining this result, it is possible to define which p value obtained with the independent *t* test, which compares means, will be used, since the p value is calculated automatically for both hypotheses of the Levene test. If the *t* test p value is > 0.05, the means for the two groups are statistically equal, i.e. there is no significant difference between them, indicating that the risk factor analyzed is not so relevant to AAA diameter. However, if p is < 0.05, the means for the two groups are statistically different, i.e., there is a significant difference between them, indicating that the risk factor analyzed has great relevance for AAA diameter (95% confidence).

The minimum sample size for each comparison between exposed and unexposed groups using Student’s *t* test was calculated with the G Power program, using a 0.05 significance level, 0.95 test power, and Cohen’s effect size of 0.80, correcting the sample size calculation for the proportion of the sample allocated to each of the two groups being compared (Table 1).

**Table 1:** Minimum sample size (corrected for the proportion of the sample allocated to each subset for the independent variables compared).

Independent variables compared	Minimum size for group 1 (corrected value)	Minimum size for group 2 (corrected value)
Group 1: DM	25 (89)	55 (191)
Group 2: no DM		
Group 1: no SAH	19 (29)	167 (253)
Group 2: SAH		
Group 1: no DLP	21 (71)	69 (209)
Group 2: DLP		
Group 1: current smokers	31 (63)	41 (84)
Group 2: never smoked		
Group 1: current smokers	26 (63)	52 (129)
Group 2: ex-smokers		
Group 1: never smoked	29 (84)	45 (129)
Group 2: ex-smokers		
Group 1: ≤ 65 years of age	32 (93)	44 (119)
Group 2: 66 to 75		
Group 1: ≤ 65 years of age	36 (93)	34 (87)
Group 2: > 75 years old		
Group 1: > 75 years old	30 (87)	42 (119)
Group 2: 66 to 75		

DM = diabetes mellitus; SAH = systemic arterial hypertension; DLP = dyslipidemia.

Additionally, a multivariate regression was conducted to investigate the combined influence of risk factors on abdominal diameter. In this step, groups of risk factors were considered simultaneously, enabling the effect of interactions between them on aneurysm diameter to be evaluated. Maintaining a 95% confidence interval, regression coefficients were estimated for each risk factor, enabling a more comprehensive understanding of the role played by each variable on aneurysmal diameter based on the p value.

In order to trace the epidemiological profile of the patients seen at the Vascular and Endovascular Surgery Department, the variables extracted from the patient records were tabulated and analyzed individually for each of the factors listed above. The distributions of means were expressed in boxplots for diabetics versus non-diabetics, hypertensives versus normotensives, patients with dyslipidemia versus those without, and smokers versus ex-smokers and versus non-smokers.

## RESULTS

### Descriptive analysis

From a total of 299 patients seen from 2007 to 2020, 232 were male (76.11%) and 67 were female (23.89%), indicating a greater prevalence among men (in addition to a greater mean aneurysm diameter: 6.580 cm for men and 6.725 cm for women). The mean of age of patients was 69.5351 years.

Of the 299 patients seen, 86 were from São Paulo city (São Paulo [SP] state), 84 were from other towns in SP, 121 were from states other than SP, and 8 patients were from abroad. There was great dispersion in terms of place of birth and, except for the city of São Paulo, no other location stood out. The dispersion of place of origin was concentrated in SP state and no patients had come from beyond SP. With relation to occupation, the great majority were retired (237), although there were also many housekeepers/housewives (27).

A total of 143 patients had been diagnosed as an incidental finding and abdominal pains, with or without swelling/pulsating mass, were the most common symptom, presented by 103 patients. Chest pains were observed in 12 patients and lumbar pain was present in 17. It was notable that 24 cases were urgent because of aneurysms in expansion or ruptured.

**Figure 2:**
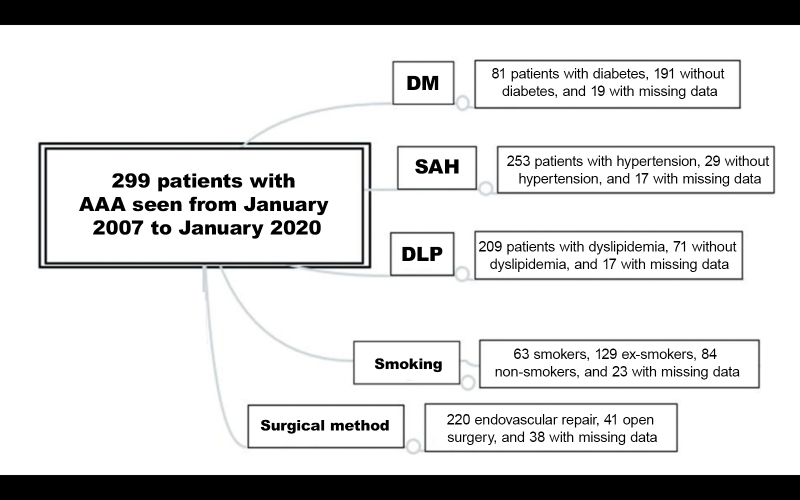
General analysis of all patients enrolled in the study: overview of surgical method employed and comorbidities (DM, SAH, DLP, and smoking). AAA = abdominal aortic aneurysm; DM = diabetes mellitus; SAH = systemic arterial hypertension; DLP = dyslipidemia.

Some patients had missing data related to risk factors, aneurysm characteristics, and procedures performed and were removed from the counts (Figure 2).

With regard to postoperative repercussions, there were 31 deaths related to AAA. Mortality was 19.04% for open surgery (8 deaths), while endovascular mortality was 3.63% (8 deaths), while for the remaining 15 deaths the patient records did not provide information on surgical method used and these cases were therefore excluded from this analysis.

### Diabetes mellitus

A subsample of 280 patients with AAA was split into two groups (19 patients did not have data on DM and were excluded from this analysis): those with DM (n = 89) and those without DM (n = 191). Calculating the mean AAA diameter per group, patients with DM had a mean diameter of 6.6242 cm, compared to 6.4277 cm for those without DM. Student’s *t* test returned a p value of 0.328. It is therefore inferred that DM was not a statistically relevant factor in AAA diameter.

**Figure 3:**
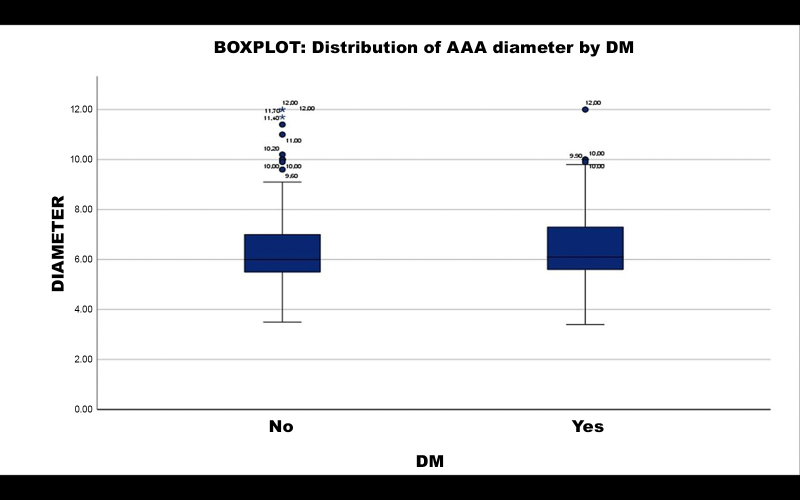
Distribution of aneurysm diameter by DM. Boxplot of distribution of aneurysm diameter by DM: patients without diabetes (NO) and patients with diabetes (YES). The group without diabetes was associated with very large AAA diameter (beyond the upper limit). AAA = abdominal aortic aneurysm; DM = diabetes mellitus.

Distribution of aneurysm diameter is illustrated in a boxplot in Figure 3.

### Arterial hypertension

A subsample of 282 patients with AAA was split into two groups (17 patients did not have data on SAH and were excluded from this analysis): those with SAH (n = 253) and those without SAH (n = 29). Calculating the mean AAA diameter per group, patients with SAH had a mean diameter of 6.5346 cm, compared to 6.3207 cm for those without SAH. Student’s *t* test returned a p value of 0.494. It is therefore inferred that SAH was not a statistically relevant factor in AAA diameter.

**Figure 4:**
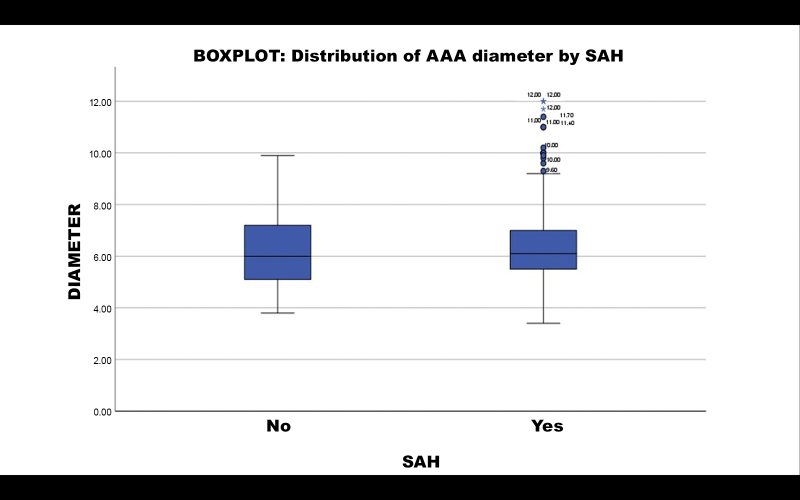
Distribution of aneurysm diameter by SAH. Boxplot of distribution of aneurysm diameter by SAH: patients without hypertension (NO) and patients with hypertension (YES). The group with hypertension was associated with very large AAA diameter (beyond the upper limit). AAA = abdominal aortic aneurysm; SAH = systemic arterial hypertension

Distribution of aneurysmal diameter is illustrated in a boxplot in Figure 4.

### Dyslipidemia

A subsample of 280 patients with AAA was split into two groups (19 patients did not have data on DLP and were excluded from this analysis): those with DLP (n = 209) and those without DLP (n = 71). Calculating the mean AAA diameter per group, patients with DLP had a mean diameter of 6.4026 cm, compared to 6.7352 cm for those without DLP. Student’s *t* test returned a p value of 0.121. It is therefore inferred that DLP was not a statistically relevant factor in AAA diameter.

**Figure 5:**
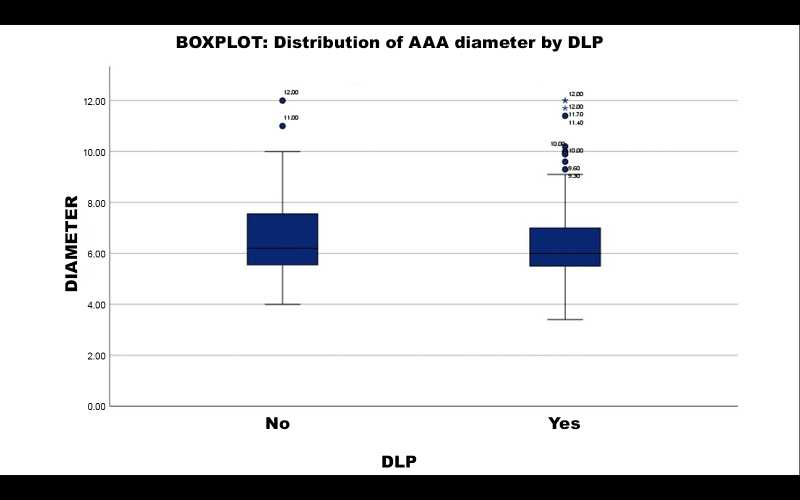
Distribution of aneurysm diameter by DLP. Boxplot of distribution of aneurysm diameter by DLP: patients without hypertension (NO) and patients with hypertension (YES). The group with dyslipidemia was associated with very large AAA diameter (beyond the upper limit). AAA = abdominal aortic aneurysm; DLP = dyslipidemia.

Distribution of aneurysmal diameter is illustrated in a boxplot in Figure 5.

### Smoking

A subsample of 276 patients with AAA was split into three groups (19 patients did not have data on smoking and were excluded from this analysis): current smokers (n = 63), those who had never smoked (n = 84), and ex-smokers (n = 129). Calculating the mean AAA diameter per group, current smokers had a mean diameter of 7.190 cm, compared to 6.3296 cm for those who had never smoked. Student’s *t* test returned a p value of 0.002. It is therefore inferred that smoking was a statistically relevant factor in greater AAA diameter among these patients compared to patients who had never smoked.

The same subset of current smokers was compared to the subset of ex-smokers, who had a mean diameter of 6.3221 cm. Student’s *t* test returned a p value of p < 0.001. It is therefore inferred that smoking was a statistically relevant factor in greater AAA diameter among these patients compared to ex-smokers.

Finally, the same comparison was made between the subsets of ex-smokers and patients who had never smoked. Student’s *t* test returned a p value of p = 0.976. It is therefore inferred that being an ex-smoker, when compared with never having smoked, was not a statistically relevant factor in AAA diameter.

**Figure 6:**
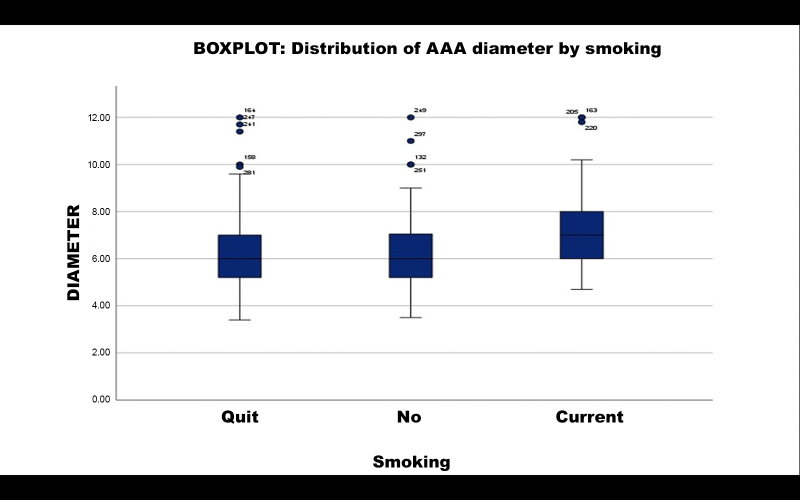
Distribution of aneurysm diameter by smoking. Boxplot of distribution of aneurysm diameter by smoking: ex-smokers, non-smokers, and current smokers. The group of current smokers was associated with very large AAA diameter (beyond the upper limit). AAA = abdominal aortic aneurysm.

Distribution of aneurysmal diameter is illustrated in a boxplot in Figure 6.

### Age

The sample of 299 patients with AAA was split into three groups: patients aged ≤ 65 years (n = 93), patients aged 66 to 75 years (n = 119), and patients aged > 75 years (n = 87). Calculating the mean AAA diameter per group, patients aged ≤ 65 years had mean diameter of 6.8365 cm, while patients aged 66 to 75 years had mean diameter of 6.3617 cm. Student’s *t* test returned a p value of 0.0226. It is therefore inferred that age ≤ 65 years was a statistically relevant factor in greater AAA diameter among these patients compared to patients aged 66 to 75 years of age.

The same patients aged ≤ 65 years were compared with the subset of patients aged > 75 years, who had a mean aneurysm diameter of 6.4758 cm. Student’s *t* test returned a p value of 0.0843. It is therefore inferred that age ≤ 65 years, when compared with age > 75 years, was not a statistically relevant factor in greater AAA diameter.

**Figure 7:**
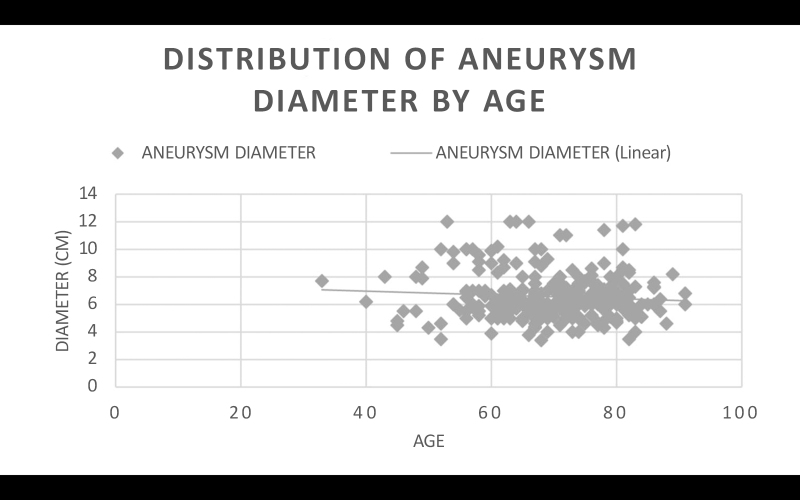
Distribution of aneurysm diameter by age: the trend line shows aneurysm diameter reducing with age. AAA = abdominal aortic aneurysm.

Finally, the same comparison was made between the subsets of patients aged 66 to 75 years and aged > 75 years. Student’s *t* test returned a p value of 0.2979. It is therefore inferred that age ≤ 65 years, when compared with age > 75 years was not a statistically relevant factor in AAA diameter (Figure 7).

### Multivariate linear regression analysis

A multivariate regression analysis was conducted with data from a total of 271 patients (18 patients were excluded from this analysis because they did not have data on all of the risk factors analyzed simultaneously). The results returned a coefficient of determination of 0.0608, which indicates that 6.08% of variance in aneurysm diameter can be explained by the independent variables included in the model. Even when the impact of all other factors was considered in the analysis, the factor current smoking remained statistically relevant to greater aneurysm diameter, when compared to ex-smokers (p = 0.020) and to patients who had never smoked (p = 0.023). The same was true of the relationship between patients aged ≤ 65 years and patients aged 66 to 75 years (p = 0.037) (Table 2).

**Table 2:** Multivariate linear regression analysis – aneurysm diameter.

Predictor	Regression coefficient estimate	Standard error	*t*	p
Reference level	7.262	0.271	26.752	< 0.001
DM:	-0.252	0.207	-1.220	0.224
N – Y				
SAH:	-0.183	0.315	-0.580	0.563
N – Y				
Smoking:	-0.606	0.265	-2.290	0.023
N – Y				
E – Y	-0.571	0.244	-2.338	0.020
N – E	-0.0353	0.221	-0.160	0.873
DLP:	0.369	0.222	1.663	0.097
N – Y				
Age:	-0.469	0.224	-2.096	0.037
66-75 – < 65				
> 75 – < 65	-0.172	0.255	-0.675	0.500
> 75 – 66-75	0.2971	0.239	1.244	0.215

Multivariate linear regression model coefficients using aneurysm diameter as dependent variable and the factors DM, SAH, smoking, DLP, and age for 271 patients. Y = has risk factor; N = does not have risk factor; E = ex-smoker; DM = diabetes mellitus; SAH = systemic arterial hypertension; DLP = dyslipidemia.

## DISCUSSION

The results support the inference that, of all the risk factors already known for development of AAA, only current smoking was relevant to greater diameter of the aneurysm sac when compared to patients who had quit smoking or had never smoked, as is observed in the literature.^35,36^ Greater diameters are related to both higher mortality secondary to ruptured aneurysms,^37^ and to higher risk of cardiovascular mortality unrelated to the aneurysm.^38^

The other known risk factors, SAH and DLP, did not demonstrate an influence on mean AAA diameter. Finally, DM also failed to demonstrate any effect on aneurysm diameter.

However, it was observed that smokers, non-diabetics, and patients with hypertension and dyslipidemia had a higher prevalence of very large diameter AAA (discrepant values beyond the upper limit of the standard deviation), when compared to unexposed groups.

With relation to the factor age, taking into account the fact that there is a prominent increase in the incidence of AAA after 65 years of age,^39,40^ the statistically relevant difference between AAA diameter of patients aged ≤ 65 years and those aged 66 to 75 years suggests that younger patients exhibit greater progression of aneurysm sac expansion. The absence of a statistical difference between the younger patients and those over the age of 75 years may be because of the natural progression of the disease, i.e. the older patients who developed AAA between 65 and 75 years of age will develop a larger aneurysm diameter over time, thereby compensating for the difference in diameter compared to younger patients.

However, it is important to recognize that the coefficient of determination returned by the multivariate analysis was relatively low (0.068), suggesting that other factors that are not included in the model may also contribute to AAA diameter variance. As such, future studies with a larger scope and inclusion of a wider range of variables may offer a more complete understanding of the determinants of AAA and its progression.

Finally, it was observed that there was increasing adoption of endovascular procedures for treatment of this pathology, rather than open surgery, which had a higher mortality rate.

The sample of patients treated at this hospital included a much greater proportion of patients with SAH and DLP than of patients who did not have these diseases. This was expected, since in the literature both these pathologies are known to be risk factors for development of AAA. In contrast, there was a lower prevalence of AAA patients with DM than of patients without DM. Patients who have smoked at some point in life (current smokers plus ex-smokers) were the majority, which is also in line with the literature. With regard to risk factors associated with AAA, these patients were distributed in a predictable manner.

This study was limited by the use of digital patient records from a single hospital. This restricted the number of patients, both because of the absolute number of records and because of information missing from some records, which could have introduced information bias.

## CONCLUSIONS

In summary, smokers appear to be at greater risk of AAA rupture when compared to non-smokers and ex-smokers, and younger patients (aged ≤ 65 years) appear to be at greater risk than patients aged 66 to 75 years of age. However, it is important to point out that the multivariate analysis coefficient of determination was relatively low, suggesting the existence of other factors not considered in this model that may influence AAA diameter. As such, studies with larger samples and a more comprehensive approach could assess influences better and are needed to supplement understanding of the determinants of aneurysm diameter.

While this study has its limitations, the results provide important information about the relationships between risk factors and AAA diameter, contributing to understanding and management of this clinical condition.
