# Supplementary material for: The relationship between abdominal aortic aneurysm diameter and its risk factors: a retrospective cohort study
Source: J Vasc Bras. 2025 Feb 14;24:e20230110. doi: 10.1590/1677-5449.202301102 (PMC11841612; doi:10.1590/1677-5449.202301102)
Supplement: Translation

# Translation: Relação entre o diâmetro do aneurisma de aorta abdominal e seus fatores de risco: uma coorte retrospectiva

**DOI:** 10.1590/1677-5449.202301101

**Authors:** João Victor Domiciano Martins, Rodrigo Mendes, Ronald Luiz Gomes Flumignan, Luiz Carlos Uta Nakano, Jorge Eduardo de Amorim, Henrique Jorge Guedes

**Affiliations:** 1 Universidade Federal de São Paulo – UNIFESP, São Paulo, SP, Brasil.; 2 Irmandade da Santa Casa de Misericórdia de São Paulo – FCMSCSP, São Paulo, SP, Brasil.

**Keywords:** aneurisma de aorta abdominal, fatores de risco, diâmetro

## Abstract

**Contexto:**

O aneurisma de aorta abdominal é definido como uma dilatação focal e persistente da porção abdominal da aorta ≥ 50% do diâmetro dos segmentos adjacentes, envolvendo as três camadas do vaso.

**Objetivos:**

Avaliar como os fatores de risco (diabetes melito, hipertensão arterial, dislipidemia, tabagismo e idade) influenciam na expansão do diâmetro aneurismático.

**Métodos:**

Trata-se de um estudo observacional retrospectivo de uma série de casos que incluiu 299 pacientes atendidos entre janeiro de 2007 e janeiro de 2020, divididos entre os grupos de expostos e não expostos aos fatores de risco. Utilizou-se o teste *t* de Student para avaliar a relação com o diâmetro aneurismático. Ademais, foi realizada uma análise de regressão multivariada com esses grupos.

**Resultados:**

Fumantes demonstraram aneurismas maiores em comparação aos que nunca fumaram (p = 0,002) e aos ex-fumantes (p < 0,01), assim como pacientes com idade ≤ 65 anos em comparação aos pacientes entre 66 e 75 anos (p = 0,0226). Não houve correlação significativa com os demais fatores de risco (diabetes melito, dislipidemia e hipertensão). A análise de regressão multivariada confirmou o mesmo resultado, porém com um coeficiente de determinação de 0,0608. Além disso, pacientes tabagistas, hipertensos, dislipidêmicos e não diabéticos apresentaram maiores frequências de diâmetros muito elevados.

**Conclusões:**

Observou-se que idade ≤ 65 anos e tabagismo atual estão relacionados com maiores diâmetros aneurismáticos. Apesar da mesma relação estatística não ter sido provada acerca de hipertensão, ausência de diabetes melito e dislipidemia, houve uma maior frequência de valores discrepantes para esses grupos. São necessários estudos com uma compreensão mais abrangente dos determinantes do diâmetro aneurismático.

## INTRODUÇÃO

O aneurisma de aorta abdominal (AAA) é uma doença degenerativa multifatorial^1-3^ , com incidência de, aproximadamente, 5% da população sem comorbidades, tornando-se mais frequente com o envelhecimento e a presença de comorbidades, como aterosclerose, hipertensão arterial sistêmica (HAS)^4-8^ e doenças do tecido conjuntivo e do colágeno^9-11^ . Ademais, há uma notável relação entre a incidência de aneurismas e tabagismo, sedentarismo e dieta hiperlipídica^7,8,12-15^ . O diabetes melito (DM) não está relacionado a um maior risco de AAA^16^ , apesar de haver controvérsias na literatura e de já ter sido considerado fator protetor anteriormente^12^ . Ademais, alguns estudos recentes sugerem que a metformina tem um papel de redução de prevalência e expansão do saco aneurismático^17^ . Aproximadamente, 175.000 mortes por ano são atribuídas a AAAs rotos^18^ .

O aneurisma de aorta pode ser definido como uma dilatação maior que 50% do diâmetro natural da aorta^19^ . A dilatação decorre da desestabilização inflamatória da camada média, que perde elasticidade e resistência por um fenômeno inflamatório denominado degeneração cística medial^20-22^ . A alteração da camada medial, associada ao fluxo sanguíneo pulsátil e em alta pressão, leva à dilatação gradual e concêntrica da aorta, formando o aneurisma^19,23-25^ .

O AAA pode manifestar-se ambulatorialmente (sintomas leves ou, majoritariamente, assintomáticos, como achado de exame^17^ ) ou como uma emergência, apresentando dor abdominal, abdome agudo hemorrágico ou choque hipovolêmico, quando há ruptura do aneurisma^19,26,27^ . O diagnóstico pode ser feito por ultrassonografia, ressonância magnética ou angiotomografia (padrão-ouro)^19,28^ . O tratamento varia de acordo com a apresentação clínica: para casos com baixo risco de rotura, são recomendados acompanhamentos regulares associados ao tratamento de doenças de base e controle de fatores de risco. A correção cirúrgica será indicada quando a dilatação for maior que 5 cm em mulheres e 5,5 cm em homens, assim como em casos de rápida expansão aneurismática^29^ . Na urgência, o tratamento baseia-se no controle do choque e cirurgia^29^ .

A cirurgia pode ser realizada pela técnica aberta, com ressecção da porção aneurismática da aorta e substituição por enxerto vascular, ou pela técnica endovascular, com colocação de endoprótese^30,31^ . Inúmeras complicações podem ocorrer dependendo da técnica cirúrgica adotada, experiência do cirurgião, posição do aneurisma, sua extensão, entre outros fatores, sendo necessário um manejo individualizado^32-34^ .

A prevalência estimada de AAA na população com idade > 50 anos da cidade de São Paulo varia entre 1,9 e 2,96%^34^ . Segundo o Instituto Brasileiro de Geografia e Estatística (IBGE), a cidade de São Paulo apresentava, em 2010, aproximadamente 6,8 milhões de habitantes com idade > 50 anos. Sendo assim, o número de portadores de AAA variava entre 129.000 e 201.000 pessoas nessa faixa etária.

Para estabelecer políticas públicas de saúde, é importante conhecer a prevalência de aneurismas de aorta em determinada população e seus subgrupos para priorizar populações de risco. O objetivo deste estudo foi determinar a relevância estatística dos fatores de risco para expansão dos AAAs em um grupo de pacientes de um hospital universitário da cidade de São Paulo. O artigo foi estruturado com base nas diretrizes Strengthening the Reporting of Observational studies in Epidemiology (STROBE) para estudos coorte.

## MATERIAIS E MÉTODOS

### Fonte de dados

Este estudo coorte retrospectivo utilizou os dados do banco de dados de prontuários digitais do hospital terciário de ensino na maior metrópole da América Latina. O estudo foi avaliado e aprovado pelo comitê de ética do hospital, Certificado de Apresentação de Apreciação Ética 46678320.3.0000.5505, Parecer Consubstanciado de número 4.869.330.

### Seleção de pacientes

Foram extraídos os dados dos prontuários dos pacientes com indicação cirúrgica de correção de AAA admitidos na Disciplina de Cirurgia Vascular e Endovascular do Hospital São Paulo entre janeiro de 2007 e janeiro de 2020. Entre as indicações cirúrgicas, foram selecionados pacientes com aneurismas rotos, sintomáticos, com rápido crescimento do diâmetro aneurismático (0,5 cm em 6 meses ou 1 cm em 1 ano), saculares e com diâmetros elevados (> 5 cm para mulheres e > 5,5 cm para homens). Entretanto, 220 prontuários eletrônicos foram excluídos devido à impossibilidade de acessar imagens para aferir o diâmetro do aneurisma e/ou à ausência de dados clínicos suficientes para as análises deste trabalho. Isto ocorreu porque parte dos prontuários de 2007 a 2011 não foram corretamente transferidos ao sistema eletrônico do hospital, implicando em um índice de perdas de 65,625% relativo ao período, enquanto o total de perdas de 2012 em diante correspondeu a 13,41% do total de prontuários (7,35% de perdas decorrentes de ausência de informações sobre os fatores de risco analisados e 6,06% decorrente da impossibilidade de acessar as imagens a fim de aferir o diâmetro aneurismático). Ademais, o processo de solicitação dos prontuários físicos impossibilitaria logisticamente a realização do trabalho em tempo hábil, visto que o processo para obtenção da autorização e requerimento desses prontuários levaria, ao menos, cerca de 6 meses. Portanto, 299 prontuários foram selecionados e analisados neste trabalho (Figura 1).

**Figura 1:** *Flowchart* demonstrando a inclusão e exclusão de pacientes. Foram admitidos 519 pacientes com aneurisma de aorta abdominal entre janeiro de 2007 e janeiro de 2020. Entre esses, 203 foram excluídos pela impossibilidade de determinar o diâmetro de seu aneurisma e 17 foram excluídos pela ausência de informações em prontuário eletrônico acerca dos fatores de risco analisados neste trabalho.

### Covariáveis e resultados

As seguintes variáveis independentes foram obtidas dos prontuários dos pacientes para qualificar a relevância dos fatores de risco: se eram ou não portadores de DM, HAS e dislipidemia (DLP), presença de tabagismo e classificação por idade (até 65 anos, entre 66 e 75 anos e acima de 75 anos). A variável dependente da análise foi o diâmetro do saco aneurismático, determinado a partir do maior diâmetro da aorta abdominal infrarrenal perpendicular ao fluxo sanguíneo, aferido a partir das tomografias de abdome acessadas pelo Synapse, um *software* conectado ao prontuário eletrônico que permite a visualização de exames de imagem.

Além disso, os seguintes fatores foram tabulados para traçar o perfil dos pacientes do hospital: sexo, ocupação, método cirúrgico (aberto ou endovascular) e procedência/naturalidade.

### Análise estatística

Os dados obtidos a partir dos prontuários foram tabulados no Excel e importados para o programa SPSS, correlacionando os diâmetros dos AAAs (variável dependente) com a existência de fatores de risco associados (variável independente), estabelecendo estatisticamente se determinado fator de risco é relevante para o maior desenvolvimento do aneurisma.

Para cada fator de risco, os pacientes foram classificados em dois grupos. Para cada grupo, o diâmetro médio foi calculado aplicando o teste de Levene, que compara variâncias, obtendo um primeiro valor de p. Se p > 0,05, assume-se que as variâncias são homogêneas (hipótese H_0_); se p < 0,05, assume-se que as variâncias não são homogêneas (hipótese H_1_).

A partir desse resultado, é possível definir qual valor de p obtido pelo teste *t* independente, que compara médias, será utilizado, visto que o valor de p é calculado automaticamente para as duas hipóteses do teste de Levene. Se o valor de p encontrado no teste *t* for > 0,05, as médias encontradas nos dois grupos são estatisticamente iguais, portanto não há diferença significativa entre elas, o que indicaria que o fator de risco analisado não tem tanta relevância no diâmetro do AAA. Porém, se p for < 0,05, as médias encontradas nos dois grupos são estatisticamente diferentes, ou seja, há diferença significativa entre elas, o que indicaria que o fator de risco analisado tem grande relevância no diâmetro do AAA (95% de confiança).

O tamanho mínimo de amostra para cada comparação entre os grupos exposto e não exposto através do teste *t* de Student foi calculado pelo programa G Power, utilizando um nível de significância de 0,05, poder do teste de 0,95 e tamanho do efeito Cohen de 0,80, corrigindo o cálculo da amostra pela proporção de alocação entre os dois grupos comparados (Tabela 1).

**Tabela 1:** Tamanho mínimo da amostra (corrigido pela proporção de alocação para cada grupo de variáveis independentes comparadas).

Variáveis independentes comparadas	Tamanho mínimo para o grupo 1 (valor corrigido)	Tamanho mínimo para o grupo 2 (valor corrigido)
Grupo 1: DM	25 (89)	55 (191)
Grupo 2: ausência DM		
Grupo 1: ausência de HAS	19 (29)	167 (253)
Grupo 2: HAS		
Grupo 1: ausência de DLP	21 (71)	69 (209)
Grupo 2: DLP		
Grupo 1: fumantes atuais	31 (63)	41 (84)
Grupo 2: nunca fumaram		
Grupo 1: fumantes atuais	26 (63)	52 (129)
Grupo 2: ex-fumantes		
Grupo 1: nunca fumaram	29 (84)	45 (129)
Grupo 2: ex-fumantes		
Grupo 1: ≤ 65 anos	32 (93)	44 (119)
Grupo 2: de 66 até 75 anos		
Grupo 1: ≤ 65 anos	36 (93)	34 (87)
Grupo 2: > 75 anos		
Grupo 1: > 75 anos	30 (87)	42 (119)
Grupo 2: de 66 até 75 anos		

DM = diabetes melito; HAS = hipertensão arterial sistêmica; DLP = dislipidemia.

Ademais, foi conduzida uma regressão multivariada para investigar a influência conjunta dos fatores de risco no diâmetro abdominal. Nessa etapa, os grupos de fatores de risco foram considerados simultaneamente, permitindo avaliar interações entre eles quanto ao diâmetro do aneurisma. Mantendo um intervalo de confiança de 95%, os coeficientes de regressão foram estimados para cada fator de risco, possibilitando uma compreensão mais abrangente do papel de cada variável no diâmetro aneurismático a partir do valor de p.

Para determinar o perfil epidemiológico dos pacientes atendidos na Disciplina de Cirurgia Vascular e Endovascular, os resultados obtidos a partir dos prontuários de cada paciente foram tabulados e contabilizados individualmente para cada fator listado acima. A distribuição das médias em forma de *boxplot* foi relatada entre diabéticos e não diabéticos, hipertensos e normotensos, dislipidêmicos e não dislipidêmicos, tabagistas, ex-tabagistas e não tabagistas.

## RESULTADOS

### Análise descritiva

De um total de 299 pacientes cadastrados entre 2007 e 2020, 232 eram do sexo masculino (76,11%) e 67 do sexo feminino (23,89%), indicando maior prevalência em homens (além de maior diâmetro médio do aneurisma: 6,580 cm para homens e 6,725 cm para as mulheres). Além disso, a média de idade dos pacientes foi de 69,5351 anos.

Dos 299 pacientes, 86 eram naturais de São Paulo (SP), 84 das demais cidades do estado, 121 de fora de SP e 8 pacientes naturais de fora do país. Há uma grande dispersão quanto ao local de nascimento dos pacientes, exceto pela cidade de São Paulo, não sendo possível destacar outro. Já em relação ao local de procedência, há uma dispersão concentrada no estado de São Paulo, sem que haja nenhum paciente vindo de fora do estado. Em relação à ocupação, a grande maioria são aposentados (237), destacando-se também as diaristas/donas de casa (27).

Entre os pacientes, 143 foram diagnosticados devido ao achado incidental de AAA, sendo a dor abdominal, acompanhada ou não de abaulamento/massa pulsátil, o sintoma mais comum, apresentado por 103 pacientes. Dor torácica também foi observada em 12 pacientes e dor lombar em 17. Ressalta-se também 24 casos relatados de aneurismas em expansão de urgência/rotura.

Sobre os fatores de risco associados, características do aneurisma e procedimento realizado, houve falta de dados em alguns pacientes, que foram retirados da contagem (Figura 2).

**Figura 2:** Análise geral de todos os pacientes incluídos neste estudo: visão geral do método cirúrgico aplicado e comorbidades (DM, HAS, DLP e tabagismo). AAA = aneurisma de aorta abdominal; DM = diabetes melito; HAS = hipertensão arterial sistêmica; DLP = dislipidemia.

Sobre as repercussões pós-operatórias, tivemos 31 óbitos relatados. A mortalidade para cirurgia aberta foi de 19,04% (8 óbitos), enquanto para cirurgia endovascular foi de 3,63% (8 óbitos), sendo que os demais 15 óbitos foram de pacientes cujos prontuários não continham informações sobre o método cirúrgico utilizado e, portanto, foram excluídos dessa contagem.

*Diabetes melito*

Separou-se, em dois grupos, 280 pacientes com AAA (19 pacientes não tinham dados sobre DM e foram excluídos desta análise): aqueles com DM (n = 89) e aqueles sem DM (n = 191). Calculando o diâmetro médio do AAA para cada grupo, os pacientes com DM apresentaram média de 6,6242 cm, enquanto os sem DM, 6,4277 cm. Aplicando o teste *t* de Student, obteve-se um valor de p = 0,328. Assim, infere-se que o fator DM não influenciou o diâmetro do AAA de forma estatisticamente relevante.

A distribuição do diâmetro aneurismático no gráfico *boxplot* desses pacientes seguiu desta forma, conforme Figura 3.

**Figura 3:** Distribuição do diâmetro do aneurisma por DM. Distribuição do diâmetro do aneurisma por DM em um gráfico de *boxplot*: pacientes não diabéticos (NÃO) e pacientes diabéticos (SIM). O grupo não diabético esteve relacionado ao diâmetro muito elevado do AAA (acima do limite superior). AAA = aneurisma de aorta abdominal; DM = diabetes melito.

### Hipertensão arterial

Separou-se, em dois grupos, 282 pacientes com AAA (17 pacientes não tinham dados sobre HAS e foram excluídos desta análise): aqueles com HAS (n = 253) e aqueles sem HAS (n = 29). Calculando o diâmetro médio do AAA para cada grupo, os pacientes com HAS tiveram média de 6,5346 cm, enquanto os pacientes sem HAS, 6,3207 cm. Aplicando o teste *t* de Student, obteve-se um valor de p = 0,494. Assim, infere-se que o fator HAS não influenciou o diâmetro do AAA de forma estatisticamente relevante.

A distribuição do diâmetro aneurismático no gráfico *boxplot* desses pacientes seguiu desta forma, conforme Figura 4.

**Figura 4:** Distribuição do diâmetro do aneurisma por HAS. Distribuição do diâmetro do aneurisma por HAS em um gráfico de *boxplot*: pacientes não hipertensos (NÃO) e pacientes hipertensos (SIM). O grupo hipertenso esteve relacionado ao diâmetro muito elevado do AAA (acima do limite superior). AAA = aneurisma de aorta abdominal; HAS = hipertensão arterial sistêmica.

### Dislipidemia

Separou-se, em dois grupos, 280 pacientes com AAA (19 pacientes não tinham dados sobre DLP e foram excluídos desta análise): aqueles com DLP (n = 209) e aqueles sem DLP (n = 71). Calculando o diâmetro médio do AAA para cada grupo, os pacientes com DLP tiveram média de 6,4026 cm, enquanto os sem DLP, 6,7352 cm. Aplicando o teste *t* de Student, obteve-se um valor de p = 0,121. Assim, infere-se que o fator DLP não influenciou o diâmetro do AAA de forma estatisticamente relevante.

A distribuição do diâmetro aneurismático no gráfico *boxplot* desses pacientes seguiu desta forma, conforme Figura 5.

**Figura 5:** Distribuição do diâmetro do aneurisma por DLP em um gráfico *boxplot*: pacientes não dislipidêmicos (NÃO) e dislipidêmicos (SIM). O grupo dislipidêmico foi relacionado ao diâmetro muito elevado do AAA (acima do limite superior). AAA = aneurisma de aorta abdominal; DLP = dislipidemia.

### Tabagismo

Separou-se, em três grupos, 276 pacientes com AAA (23 pacientes não tinham dados sobre tabagismo e foram excluídos desta análise): fumantes atuais (n = 63), aqueles que nunca fumaram (n = 84) e ex-fumantes (n = 129). Calculando o diâmetro médio do AAA para cada grupo, os fumantes atuais apresentaram uma média de 7,1905 cm, enquanto os pacientes que nunca fumaram, 6,3296 cm. Aplicando o teste *t* de Student, obteve-se um valor de p = 0,002. Assim, infere-se que o fator tabagismo atual influenciou de forma estatisticamente relevante para um diâmetro maior nesses pacientes quando comparados aos pacientes que nunca fumaram.

Os mesmos fumantes atuais foram comparados ao grupo de ex-fumantes, sendo que estes apresentaram diâmetro médio de 6,3221 cm. Aplicando o teste *t* de Student, obteve-se um valor de p < 0,001. Assim, infere-se que o fator tabagismo atual influenciou de forma estatisticamente relevante para um diâmetro maior nesses pacientes quando comparados aos ex-fumantes.

Além disso, a mesma comparação foi feita entre os grupos de ex-fumantes e pacientes que nunca fumaram. Aplicando o teste *t* de Student, obteve-se um valor de p = 0,976. Assim, infere-se que o fator ser ex-fumante, quando comparado com pacientes que nunca fumaram, não influencia de forma estatisticamente relevante o diâmetro aneurismático.

A distribuição do diâmetro aneurismático no gráfico *boxplot* desses pacientes seguiu desta forma, conforme Figura 6.

**Figura 6:** Distribuição do diâmetro do aneurisma por tabagismo em um gráfico de *boxplot*: ex-fumantes, não fumantes e fumantes atuais. O grupo de fumantes atuais foi relacionado ao diâmetro muito elevado do AAA (acima do limite superior). AAA = aneurisma de aorta abdominal.

### Idade

Separou-se, em três grupos, 299 pacientes com AAA: pacientes com idade ≤ 65 anos (n = 93), entre 66 e 75 anos (n = 119) e > 75 anos (n = 87). Calculando o diâmetro médio do AAA para cada grupo, os pacientes de até 65 anos apresentaram uma média de 6,8365 cm, enquanto os pacientes entre 66 e 75 anos, 6,3617 cm. Aplicando o teste *t* de Student, obteve-se um valor de p = 0,0226. Assim, infere-se que o fator idade ≤ 65 anos atual influenciou de forma estatisticamente relevante para um diâmetro maior nesses pacientes quando comparados aos pacientes entre 66 e 75 anos de idade.

Os mesmos pacientes com idade ≤ 65 anos foram comparados com o grupo de pacientes com idade > 75 anos, sendo que estes apresentaram um diâmetro médio de 6,4758 cm. Aplicando o teste *t* de Student, obteve-se um valor de p = 0,0843. Assim, infere-se que o fator idade ≤ 65 anos não influenciou de forma estatisticamente relevante para um diâmetro maior quando comparado a pacientes com idade > 75 anos.

Ademais, a mesma comparação foi feita entre os grupos de idade entre 66 e 75 anos e > 75 anos. Aplicando o teste *t* de Student, obteve-se um valor de p = 0,2979. Assim, infere-se que o fator idade, ao comparar esses dois grupos, não influenciou de forma estatisticamente relevante o diâmetro aneurismático (Figura 7).

**Figura 7:** Distribuição do diâmetro do aneurisma por idade: a linha de tendência demonstra uma diminuição do diâmetro do aneurisma de acordo com a idade. AAA = aneurisma de aorta abdominal.

### Análise de regressão linear multivariada

Após submeter os grupos a uma análise de regressão multivariada envolvendo um total de 271 pacientes (18 pacientes foram excluídos dessa análise por não apresentarem dados sobre todos os fatores de risco analisados simultaneamente), os resultados revelaram um coeficiente de determinação de 0,0608, o que significa que 6,08% da variabilidade do diâmetro aneurismático pode ser explicada pelas variáveis independentes incluídas no modelo. O fator tabagismo atual, mesmo ao considerar o impacto dos demais fatores na análise, manteve-se relacionado, de forma estatisticamente relevante, a maiores diâmetros aneurismáticos quando comparados a ex-tabagistas (p = 0,020) e pacientes que nunca fumaram (p = 0,023). A mesma relação manteve-se verdadeira ao comparar pacientes com idade ≤ 65 anos e pacientes entre 66 a 75 anos (p = 0,037) (Tabela 2).

**Tabela 2:** Análise de regressão linear múltipla – diâmetro do aneurisma.

Preditor	Estimativa do coeficiente de regressão	Erro-padrão	*t*	P
Nível de referência	7,262	0,271	26,752	< 0,001
DM:	-0,252	0,207	-1,220	0,224
N – S				
HAS:	-0,183	0,315	-0,580	0,563
N – S				
Tabagismo:	-0,606	0,265	-2,290	0,023
N – S				
E – S	-0,571	0,244	-2,338	0,020
N – E	-0,0353	0,221	-0,160	0,873
DLP:	0,369	0,222	1,663	0,097
N – S				
Idade:	-0,469	0,224	-2,096	0,037
66-75 – < 65				
> 75 – < 65	-0,172	0,255	-0,675	0,500
> 75 – 66-75	0,2971	0,239	1,244	0,215

Coeficientes do modelo de regressão linear múltipla utilizando o diâmetro do aneurisma como variável dependente e os fatores DM, HAS, tabagismo, DLP e idade para 271 pacientes. S = portador do fator de risco; N = não portador do fator de risco; E = ex-tabagista; DM = diabetes melito; HAS = hipertensão arterial sistêmica; DLP = dislipidemia.

## DISCUSSÃO

A partir dos resultados, pode-se inferir que, entre os fatores de risco já conhecidos para o desenvolvimento do AAA, apenas o tabagismo atual foi relevante para um maior diâmetro do saco aneurismático quando comparado aos pacientes que pararam de fumar ou nunca fumaram, assim como é bem observado na literatura^35,36^ . Maiores diâmetros estão relacionados tanto a uma maior mortalidade em decorrência de aneurismas rotos^37^ , quanto a um maior risco de mortalidade cardiovascular não aneurismática^38^ .

Os outros fatores de risco conhecidos, que são a HAS e a DLP, não demonstraram influenciar o diâmetro médio do AAA. Por fim, o DM não demonstrou o mesmo efeito quando considerado o diâmetro aneurismático.

No entanto, observa-se que, para tabagistas, não diabéticos, dislipidêmicos e hipertensos, há maior prevalência de AAA com diâmetro muito elevado (valores discrepantes, acima do limite superior do desvio-padrão) quando comparado aos grupos não expostos.

Em relação ao fator idade, tendo em vista que há um aumento proeminente da incidência do AAA após os 65 anos de idade^39,40^ , a diferença estatisticamente relevante entre os diâmetros do AAA de pacientes com idade ≤ 65 anos e aqueles entre 66 e 75 anos sugere que pacientes mais jovens apresentam uma maior progressão do saco aneurismático. A ausência de diferença estatística entre os pacientes mais jovens e aqueles com idade > 75 anos pode ser explicada pela evolução natural da doença: os pacientes mais velhos, que desenvolveram o AAA entre 65 e 75 anos, apresentarão um maior diâmetro aneurismático após a passagem do tempo, compensando, assim, a diferença do diâmetro quando comparado aos pacientes mais jovens.

Todavia, é importante reconhecer que o coeficiente de determinação obtido na análise multivariada foi relativamente baixo (0,068), o que sugere que outros fatores não incluídos no modelo podem contribuir para a variabilidade no diâmetro do AAA. Portanto, estudos futuros com uma abordagem mais abrangente e a inclusão de uma gama mais ampla de variáveis podem fornecer uma compreensão mais completa dos determinantes do AAA e de sua progressão.

Por fim, notou-se uma maior adoção de procedimentos endovasculares para o tratamento dessa patologia em detrimento das cirurgias abertas, que apresentaram maior taxa de mortalidade.

Entre os pacientes atendidos no hospital, evidenciou-se maior prevalência de pacientes com HAS e DLP quando comparados aos que não sofriam dessas doenças. Esse era um fato já esperado, pois na literatura ambas as patologias são indicadas como fatores de risco para o desenvolvimento de AAA. Os portadores de DM, por outro lado, apresentaram menor prevalência entre os pacientes com AAA quando comparados aos não portadores. Pacientes que fumaram uma vez na vida também foram mais prevalentes (adicionando fumantes atuais e ex-fumantes), também concordante com a literatura. Pode-se dizer que, em relação aos fatores de risco associados ao AAA, os pacientes estão distribuídos de forma previsível.

Este estudo foi limitado pela utilização do banco de dados de prontuários digitais de um único hospital. Por isso, a quantidade de pacientes foi restringida pelo número limitado de prontuários e pela falta de informações em alguns desses, o que poderia ser fonte de viés de informação.

## CONCLUSÃO

Em suma, fumantes parecem ter maior risco de desenvolver AAA roto quando comparados a não fumantes e ex-fumantes, assim como pacientes mais jovens (idade ≤ 65 anos) quando comparados a pacientes entre 66 e 75 anos de idade. No entanto, é importante ressaltar que o coeficiente de determinação na análise multivariada foi relativamente baixo, sugerindo a existência de outros fatores não considerados no modelo que podem influenciar no diâmetro do AAA. Portanto, estudos de maiores amostras e com uma abordagem mais abrangente poderiam avaliar melhor a influência, sendo necessários para complementar a compreensão sobre os determinantes do diâmetro aneurismático.

Embora este estudo tenha suas limitações, os resultados fornecem informações importantes sobre a relação entre fatores de risco e o diâmetro do AAA, contribuindo para a compreensão e o manejo dessa condição clínica.
